# Fold-specific sequence scoring improves protein sequence matching

**DOI:** 10.1186/s12859-016-1198-z

**Published:** 2016-08-30

**Authors:** Sumudu P. Leelananda, Andrzej Kloczkowski, Robert L. Jernigan

**Affiliations:** 1 Department of Biochemistry, Biophysics and Molecular Biology, Iowa State University, 112 Office and Lab Building, Ames, IA 50011-3020 USA; 2 Laurence H. Baker Center for Bioinformatics and Biological Statistics, Iowa State University, 112 Office and Lab Building, Ames, IA 50011-3020 USA; 3 Present Address: 2120 Newman and Wolfrom Laboratory, The Ohio State University, 100 W 18th Ave, Columbus, OH 43210 USA; 4 Present Address: Battelle Center for Mathematical Medicine, The Research Institute at Nationwide Children’s Hospital, Columbus, OH 43205 USA; 5 Present Address: Department of Pediatrics, The Ohio State University College of Medicine, Columbus, OH 43205 USA

**Keywords:** Sequence matching, Protein fold families, CATH topologies, Distant homologies, Blosom62, Structure alignment, HMM, Psi-blast

## Abstract

**Background:**

Sequence matching is extremely important for applications throughout biology, particularly for discovering information such as functional and evolutionary relationships, and also for discriminating between unimportant and disease mutants. At present the functions of a large fraction of genes are unknown; improvements in sequence matching will improve gene annotations. Universal amino acid substitution matrices such as Blosum62 are used to measure sequence similarities and to identify distant homologues, regardless of the structure class. However, such single matrices do not take into account important structural information evident within the different topologies of proteins and treats substitutions within all protein folds identically. Others have suggested that the use of structural information can lead to significant improvements in sequence matching but this has not yet been very effective. Here we develop novel substitution matrices that include not only general sequence information but also have a topology specific component that is unique for each CATH topology. This novel feature of using a combination of sequence and structure information for each protein topology significantly improves the sequence matching scores for the sequence pairs tested. We have used a novel multi-structure alignment method for each homology level of CATH in order to extract topological information.

**Results:**

We obtain statistically significant improved sequence matching scores for 73 % of the alpha helical test cases. On average, 61 % of the test cases showed improvements in homology detection when structure information was incorporated into the substitution matrices. On average z-scores for homology detection are improved by more than 54 % for all cases, and some individual cases have z-scores more than twice those obtained using generic matrices. Our topology specific similarity matrices also outperform other traditional similarity matrices and single matrix based structure methods. When default amino acid substitution matrix in the Psi-blast algorithm is replaced by our structure-based matrices, the structure matching is significantly improved over conventional Psi-blast. It also outperforms results obtained for the corresponding HMM profiles generated for each topology.

**Conclusions:**

We show that by incorporating topology-specific structure information in addition to sequence information into specific amino acid substitution matrices, the sequence matching scores and homology detection are significantly improved. Our topology specific similarity matrices outperform other traditional similarity matrices, single matrix based structure methods, also show improvement over conventional Psi-blast and HMM profile based methods in sequence matching. The results support the discriminatory ability of the new amino acid similarity matrices to distinguish between distant homologs and structurally dissimilar pairs.

**Electronic supplementary material:**

The online version of this article (doi:10.1186/s12859-016-1198-z) contains supplementary material, which is available to authorized users.

## Background

With more and more genomes being sequenced and the resulting problem of poor annotations becoming more critical it is important to turn attention to improving sequence matching to enable better identification of function. The most common way to annotate genes and identify the function of a new gene is based on identifying a similar sequence by sequence matching against proteins of known function. However, this remains a challenge [1–8], and it is generally thought that ~40 % of genes do not have a known function. Also for protein structure prediction, sequence matching of protein sequences is the standard way to identify protein homologs - the first step in protein homology model building. The huge numbers of protein sequences with unknown structures and unknown functions cannot be identified by the present sequence matching with the present sets of annotated sequences and structures. Thus, improving protein sequence matching should enable improving both the identification of remote homologs, for the predictions of the structures and function of large numbers of protein sequences.

The quality of sequence alignments and the similarity scores used for sequence matching depend critically on the amino acid substitution matrices that are used. Substitution matrices are developed by alignment of protein sequences. Alignment is important in biology and can reveal crucial information about evolution. These alignments can identify patterns of sequence conservation of proteins that belong to fold families. Although sequence alignments are popular and far more frequently performed they are not very reliable whenever sequences are too different showing little sequence similarity. These are cases sometimes called the twilight and midnight zones. There are cases where similar structures have extremely little or no sequence similarity. Moreover, there are also structures that have sequences that are very similar but have completely different folds [9]. When the sequence identity between two related proteins falls below 30 %, sequence-based search methods do not perform well [10].

However, when protein structures are more conserved than their sequences [11] it will be more reliable to align structures instead of sequences. Improved substitution matrices can be built from structure alignments, based on the amino acid identities of proximate pairs in the aligned structure pair. Better substitution matrices can capture distant evolutionary relationships and can more reliably detect distant evolutionary relationships. Success will depend on how well these newly developed matrices can detect the sequence similarities among distantly related sequences.

Amino acid substitutions do not obey universal rules. There are some amino acid substitutions that more commonly occur in related proteins. It is important for the substituted amino acids to be compatible with the protein structure and function. Some have postulated that a specific amino acid in one position is conserved for different reasons than at other positions [12–14]. If a substitution is not compatible, then a single mutation can modify the protein structure and sometimes, though rarely, even disrupt the whole protein function or denature the protein. Often, these substitutions are preferential for chemically similar or size similar amino acids or similar in charge. However, other changes may also occur to compensate for neighboring changes –so-called compensatory mutations. Residues on the surface can often be substituted with nearly any type of amino acid, unless it is a critical functional residue. If we know the specific types of changes that are most and least common within a large number of proteins, this information can assist with sequence matching. But combining information from many different types of structures means that the substitution metric loses much of its specificity. The availability of a large body of sequence and structure information can aid in expanding these types of statistical computational methods.

### Sequence-based similarity matrices

The earliest similarity matrices were the Pam matrices from from Margaret Dayhoff’s group [15], which were based upon extremely small numbers of manually aligned sequences and then the Blosum matrices [16], which were developed later using much more. These matrices are statistical matrices where the frequency of occurrence of mutations is used for their derivation. Another type of substitution matrix that is based on amino acid contact frequencies in proteins was reported by Miyazawa and Jernigan [17]. Statistical amino acid contact potential based similarity matrices have also been developed [18]. Contact propensity of amino acids is a strongly conserved feature of each position of a protein, and amino acid matrices can be obtained from the correlations within the pairwise amino acid contact potentials. Vilim et al. developed substitution matrices employing a method similar to Blosum. However, instead of focusing on positions that are strongly conserved they considered particularly positions that are different within a family of proteins [19].

There are also studies where multiple sequence information was used to develop Hidden Markov Models for families of proteins [20, 21]. In another study, Kuznetsov et al. compared general purpose matrices and found that the maximum likelihood method [22] is the best performing standard matrix [23]. However, they also stated that no generic matrix can outperform all other matrices for all protein structural folds. Some of the other similarity matrices developed in the past include those of Luthy et al., Niefind et al., Overington et al., Koshi et al., and Russell et al. [24–28]. Tomii et al. [29] obtained mutation matrices using amino acid indices which are a set of numerical values representing any of the different physicochemical and biochemical properties of amino acids. They used 42 published matrices and performed cluster analysis to construct a substitution matrix. They also tried to reproduce these starting substitution matrices by combining amino acid indices and found that matrices like PAM and volume and hydrophobicity of amino acids are correlated. In a recent study done by Yamada and Tomii [30], they developed a principal component analysis based matrix using existing substitution matrices, Blosum, VTML [22] and BCG [31]. Their results proved were improved compared to generic purpose substitution matrices.

### Structure information in similarity matrices

Since structure is more conserved than sequence [11], a more appropriate way to approach this issue is by using structure alignments. Prlic et al. used structure alignments to derive similarity matrices (PRLA1) [32]. They used a data set of superimposed protein pairs to derive evolutionary information. These pairs have high structural similarity but low sequence similarity. Structural information has also been used to enrich substitution matrices [33]. They used a linear combination of the sequence substitution matrix Blosum50 and a threading energy table Thom2. The resulting matrix was shown to improve the prediction accuracy for homology modeling in the twilight zone. It was suggested that by further incorporating protein structural descriptors such as secondary structure and exposed surface area in a linear fashion better performance could be obtained. The Johnson and Overington matrix (JOHM) takes into account not only the substitutions that occur in similar parts of protein structures but also the variable regions where gaps occur [34]. Blake and Cohen built similarity matrices (eg: BC0030) where structural superposition of protein structures was performed by using structures obtained from CATH database [35]. The structures were selected based on the sequence identities and the alignments were performed for ranges of different sequence identities. These series of matrices were used in structure-function prediction. Some studies have shown that the use of protein family specific substitution matrices is helpful to identify orthologs that are not identified with the standard Blosum matrices [36]. In that study the authors developed parasite specific similarity matrices and were thereby able to annotate apicomplexan proteins which have unknown functions.

It is clear that structure based matrices are key for improvements in sequence matching. Although there are some methods that use structure information they fail to capture the unique information for each topology. It is important that the substituted amino acids are compatible with the protein structure and function. For most protein sequence comparisons only a single amino acid matrix is used. The overall goal in this study is to develop different amino acid substitution matrices for different topologies of proteins. The novelty in our work resides in several of its aspects. We employ a novel multi-structure alignment at each homology level (H) of the CATH structures in order to extract topological information. In addition to using structure information we also use a sequence information component to find the unique evolutionary conserved residues in each CATH topology. A weighting system between these two components is used to determine the optimal portion of sequence and structure information that gives the best fit for sequence matching.

## Methods

The CATH database of the hierarchical domain classification of protein structures was utilized to obtain the structures to develop our topology-based similarity matrices. The S35 family [37] of CATH data was used where structures are clustered at the 35 % level of sequence identity. We are interested in distinctions at the topology or fold level of structures. In the fold level, structures are grouped together by the overall shape of structures and the connectivity of the secondary structure elements. One hundred ninety eight all helical topologies, 137 all beta sheet topologies and 330 alpha/beta mixed topologies were chosen (Additional file 1). We have not included topologies having little secondary structure. The amino acid compositions along with other properties of these three structural classes differ [38, 39] and therefore we wanted to treat these three classes separately.

Structures were categorized according to their CATH topology (T level). Each set of sequences that belong to the same CATH topology was further categorized into its homology level (H level). These homology level structures are categorized as sharing a common ancestor in CATH. Proteins that share the same H level are said to be evolutionarily related or homologous. Proteins belonging to different H levels are called analogous and they have no evolutionary relationships. For sequences belonging to each homology level multiple structure alignments were performed using the Mustang (MUltiple STructural AligNment AlGorithm) alignment program [40], which uses a progressive pairwise framework to build and report the multiple structure alignments and superposition of structures. At least two structures need to be available for each topology in order to perform a structure alignment. A further breakdown of topology levels into the homology level is required because the homology level structural similarity is necessary to obtain reasonably good structure alignments. Doing structure alignments at the topology level is ill-posed and not very sensible.

Mustang yields a sequence alignment based on the multiple structure alignment of each homology level structure set. A C^α^ cutoff distance was used to find the residues in each pair of structures lying close together within the alignment. If a pair of residues belonging to two different protein structures in the structure alignment were found within the cutoff distance, they were then considered to be substitutions between the two structures. This is the physical basis for our substitution matrix. If there were no amino acid pairs between a pair of structures within the cutoff distance, then this was counted as a gap in the corresponding multiple sequence alignment that is developed.

**Fig. 1:**
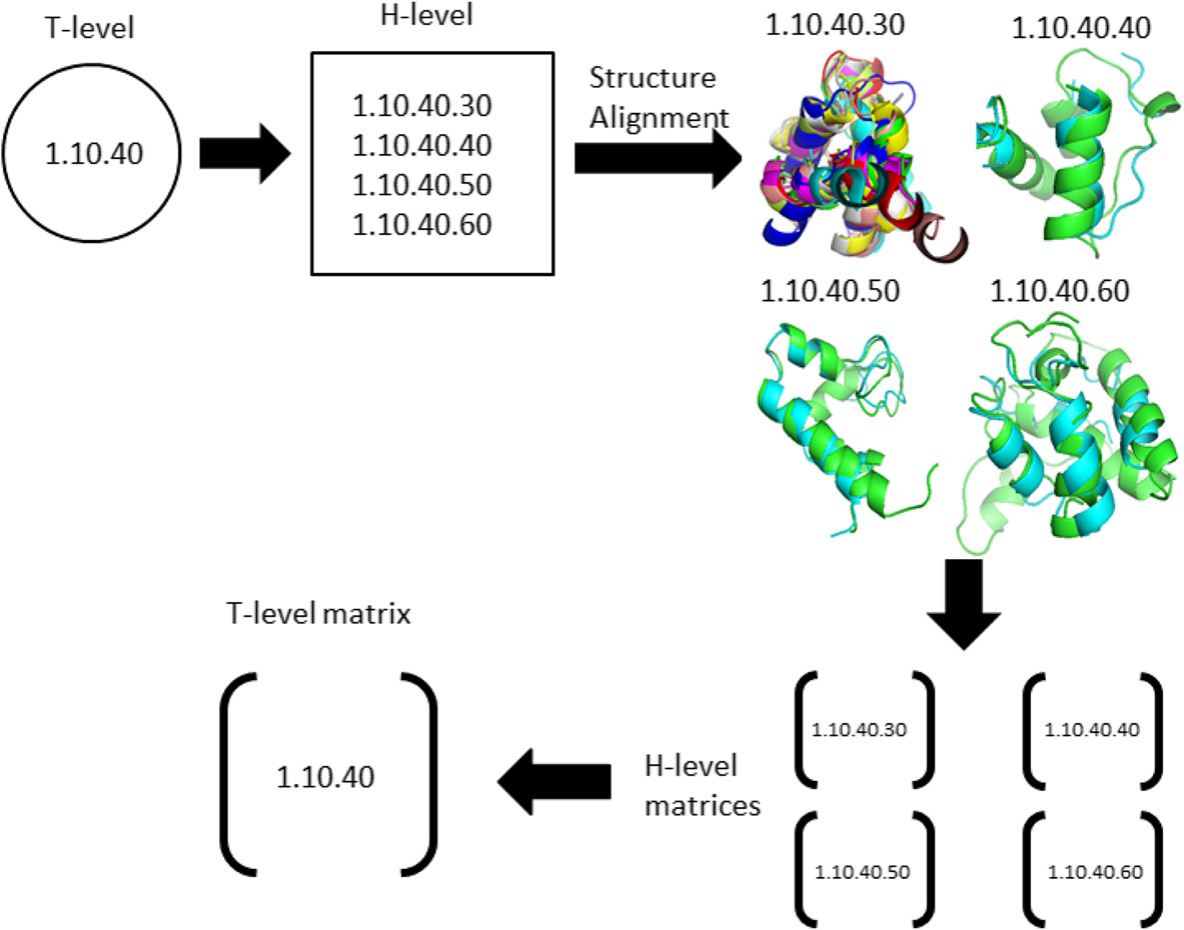
Example of how the amino acid similarity matrices are developed for one topology T-level (1.10.40). The available homology level (H-level) structure alignments are first used to obtain homology level similarity matrices. Then these homology level matrices (1.10.40.30, 1.10.40.40, 1.10.40.50, 1.10.40.60) are added together to yield the corresponding topology level matrix (1.10.40). Further decomposition of topology levels into homology level is required because homology level structural similarity is necessary to obtain reasonable structure alignments.

Eight distance cutoffs between 1 Å and 8 Å were first used for our preliminary test set. All pairwise sequence comparisons were carried out on these multiple sequence alignments and the number of times one amino acid was substituted by another was counted at each homology level. A 21×21 matrix (including gaps) was obtained containing the total number of counted substitutions. Normalization was performed by dividing the count for each type of substitution by the number of amino acids involved in that particular substitution. For example, if amino acid C was replaced with amino acid R x number of times, that count was then divided by the number of times C was replaced with any other amino acid that was not R (including C being replaced by a gap). All the homology level similarity matrices belonging to the same topology were added together in order to obtain the topology level substitution matrices. The normalized matrices were obtained by normalizing the maximum value of the 20×20 matrix to 1. A schematic diagram of how homology level matrices are added together in order to get the corresponding topology level matrix (1.10.10) is shown in Fig. 1.

The topology matrices were further modified by adding in a standard similarity matrix (Blosum62 or VT160_RA [41]). The VT160_RA matrix is based on a mathematical formalism which is referred to as the resolvent approach and protein evolution is modeled as a Markov process. This method takes into account various degrees of evolutionary divergence and iteratively cycles between estimating the evolutionary distance between an alignment and updating the estimator for the matrix.

The generic matrices were added to topology based matrices with different weight coefficients (x) varying from 1 to 30 for the topology-based matrices, to obtain the final similarity matrices that incorporate the new topology information:

$$ \mathrm{Combined}\kern0.5em \mathrm{matrix}=\mathrm{Standard}\kern0.5em \mathrm{matrix}+\mathrm{x}\left(\mathrm{Topology}-\mathrm{based}\kern0.5em \mathrm{matrix}\right). $$

In our preliminary calculation using 27 topologies with weights from 1 to 100, we observed that the best weights always lie between 10 and 30. Therefore the weight range was selected to be from 10 to 30 in the subsequent studies. These combined matrices were multiplied by 100 and round to the nearest integer in order to capture all the minor variations as well. The maximum value of the matrix was set to 20.

### Test dataset

We wanted to test our topology based matrices against a distant homologs protein set. Sets of structurally similar but sequence dissimilar pairs (SSSD) of proteins are used as the test dataset. These selected pairs have unique CATH topologies (Additional file 1).

The preliminary study was performed on a dataset consisting of 27 CATH topologies which were obtained by Friedberg et al. [42] and Prlic et al. 2000 [32]. The list of the test topologies and the sequences can be found on our website. These sequence pairs have less than 30 % sequence identity. There are eight all helical sequence pairs, nine all beta sheet sequence pairs and ten alpha/beta sequence pairs in our selected set of sequence pairs. The preliminary dataset was used to obtain the best cutoff distances for structure alignment and it also gave an indication of the relative performance from using the different standard matrices used to obtain the combined matrices. The preliminary dataset has also been used to find the appropriate range of weights to investigate.

After doing the first calculations using the above mentioned preliminary dataset, a second dataset which was larger and more complete was obtained from the GG benchmark dataset [43] for the subsequent studies. Two hundred seventy six sequences belonging to 92 unique CATH topology were extracted so that each CATH topology has three sequences. All these sequences show less than 30 % sequence identity. There are 27 all helical topologies, 19 all beta sheet and 46 alpha/beta topologies (Additional file 1). These sequences can also be downloaded from our website. Since there are three sequences for each unique topology, three sets of pairs are included for each topology. This gives three independent subsets for the second dataset. These three subsets will be referred to as test set A, test set B and test set C below. All calculations have been performed on each of these subsets, and the results are seen to agree well with each other. Results were obtained separately for each of the three classes. For each topology in each class, alignment scores were calculated for all sequence combinations using the Needleman-Wunsch algorithm from the Bioshell package [44] with default gap penalties(gap opening penalty = 10, gap extension penalty = 1). Here the goal was to learn whether the structure-based matrices are capable of distinguishing the structurally similar pairs of sequences from the other pairs better than can be done using standard generic matrices alone.

Suppose there are n number of topologies in the test set for one class and the sequences for the first topology is S_1_ and S_1_’, and that for the n^th^ topology are S_n_ and S_n_’. Then the scores are calculated for the structurally similar sequence pairs (S_1_:S_1_’, S_2_:S_2_’ … S_n_:S_n_’) and for each cross-pair that does not belong to the same topology (S_1_:S_2_’, S_1_:S_3_’…. S_1_:S_n_’; S_2_:S_1_’, S_2_:S_3_’, S_2_:S_n_’; S_n_:S_1_’, S_n_:S_n-1_’…. etc.). For topology number 1, the S_1_:S_1_’ score (which is the structurally similar score) is expected to be distinguishable from all the cross-pairs for that topology (S_1_:S_2_’, S_1_:S_3_’,…. S_1_:S_n_’).

For each topology, the scores were obtained for four matrices: the corresponding new combined topology-based matrix, the Blosum62 matrix, the VT160_RA matrix and the VTML200 matrix. VTML200 is another generic matrix that uses a maximum likelihood estimator [22]. The Blosum62 matrix does not take into account evolutionary distances; however, VT160_RA and VTML200 matrices do.

In order to compare the results obtained for different matrices, z-scores were calculated using the mean and standard deviation (SD) of data with the following equation:

$$ \mathrm{Z}-\mathrm{score}=\left(\mathrm{score}\kern0.2em \mathrm{of}\kern0.2em \mathrm{the}\kern0.2em \mathrm{structurally}\kern0.2em \mathrm{similar}\kern0.2em \mathrm{pair}\kern0.2em {\textstyle \hbox{-}}\kern0.2em \mathrm{mean}\right)/\mathrm{S}\mathrm{D}. $$

For the given example,

$$ \mathrm{Z}-\mathrm{score}=\left(\left(\mathrm{score}\kern0.2em \mathrm{of}\kern0.2em {\mathrm{S}}_1:{\mathrm{S}}_1^{\prime}\right)-\mathrm{mean}\kern0.2em \mathrm{score}\right)/\mathrm{S}\mathrm{D}. $$

Scores were obtained for all the cross set sequence pairs and structurally similar sequence pairs for all topologies of the three classes. These scores correspond to different weight coefficients. Weight 0 corresponds to the standard matrix alone (Blosum62/VT160_RA). The mean and the standard deviation for each matrix were calculated and the z-scores are obtained. After the z-scores were obtained for each weight of the corresponding combined matrix for the topology and that for Blosum62, VT160_RA and VTML200, comparisons were carried out. Then we find the similarity matrix that gave the best z-scores, that is, which matrix was able to best distinguish the structurally similar pairs from all of the other structurally dissimilar pairs. In this way we can learn which matrices are best for identifying distant homologs.

The newly developed structure based matrices were also compared with other structure based matrices in literature. As previously described z-scores were calculated for topology based matrices and other structure based matrices such as BC0030 [35], JOHM [34], and PRLA1 [32]. The performance of topology based matrices was also compared with two well established methods, Psi-blast and Hidden Markov Model (HMM) methods.

#### Psi-blast

Psi-blast is a frequently used search algorithm to detect homologous sequences [45]. For each class, the 2^nd^ sequence of the sequence matching pair for each topology (S_1_’, S_2_’, S_3_’.., S_n_’) was used to obtain the Psi-blast database for each class and was formatted for Psi-blast search. The test query sequences were S_1_, S_2_, S_3_, …, S_n_. The sequences used in obtaining the topology based matrices for each topology were used as input for each topology. The multiple sequence alignments for these sequences were obtained from clustalW. Two rounds of interactions were used. For each topology, the hits for each query sequence (S1, S2, S3, …, Sn) were obtained using the database that contain S1’, S2’, S3’.., Sn’. For each test query sequence, it was tested to see if the correct structure matching hit was identified from the database. Psi-blast was also run by replacing the default Blosum62 matrix with the corresponding topology based matrix for each case. A matrix corresponding to weight 5 was used for each case of the topology based matrix and the values in the matrix were divided by 100 and converted to the nearest integer before being used as input into Psi-blast algorithm.

#### Profile HMM

Hidden Markov Models (HMM) for sequence matching are probabilistic models that are found to be efficient for homology detection [46–48]. The sequences that were used to create each topology based matrix were used to obtain multiple sequence alignments and to generate HMM profiles for each topology. HMMER software was used for the generation of HMM profiles [49, 50]. As in Psi-blast, the 2^nd^ sequence of the sequence matching pair for each topology (S_1_’, S_2_’, S_3_’.., S_n_’) was used to obtain the Psi-blast database for each class. The database was searched using HMMER hmmsearch for each query sequence S_1_, S_2_, S_3_, …, S_n_ using the corresponding HMM profile built for each topology. Each test query sequence was tested to see if the correct structure match pair can be identified from the database.

## Results and discussion

**Fig. 2:**
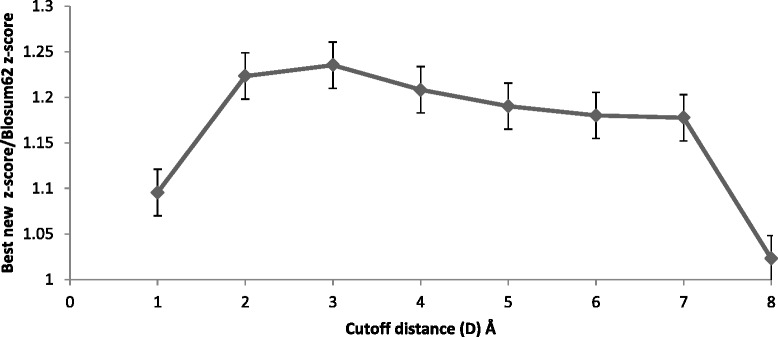
The ratio of the best average z-scores obtained with the Blosum62 combined with topology-based matrices to the Blosum62 z-score for the all helical protein set, for varying cutoff distances in the structure alignment. The z-scores are obtained for Blosum62 combined with topology-based matrices for a range of weight coefficients. The maximum (best) z-score for each topology is averaged over all helical topologies to obtain the best new z-score for the all helical class

**Fig. 3:**
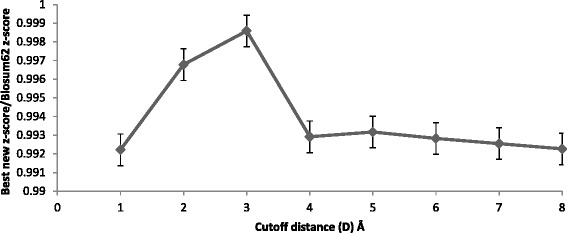
The ratio of the best average z-scores obtained for the Blosum62 combined with topology-based matrices to the Blosum62 z-score for all beta sheet protein set, for varying cutoff distances for defining the sequence match from the structure alignment. The z-scores are obtained for the Blosum62 combined topology-based matrices for a range of weight coefficients. The maximum (best) z-score for each topology is averaged over all beta sheet topologies to obtain the best new z-score for the all beta sheet class

**Fig. 4:**
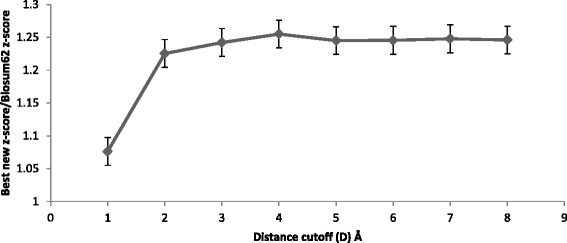
The ratio of the best average z-scores obtained with the Blosum62 combined with topology-based matrices to the Blosum62 z-score for mixed alpha and beta protein set, for varying cutoff distances for defining sequence matches. The z-scores are obtained for the Blosum62 combined with topology-based matrices for a range of weight coefficients. The maximum (best) z-score for each topology is averaged over the mixed alpha and beta topologies to obtain the best new z-score for the alpha/beta class

Figures 2, 3, and 4 show the maximum (best) average z-score obtained by topology matrices relative to Blosum62 matrix z-score for a range of structure alignment cutoff distances (D); Fig. 2 is for all helical, Fig. 3 is for all beta sheet and Fig. 4 is for alpha/beta. All three figures show a peak near 3 Å, indicating this to be an optimal distance cutoff. This peak is sharpest for the all beta sheet case although the variation is small. For alpha/beta case the decrease in the z-scores is small and remains nearly constant. From the figures the best distances are chosen to be three, 3 and 4 Å for all helical, all beta sheet and alpha/beta respectively. Although for alpha/beta, the choice between 3 and 4 Å cutoff distances do not make a significant difference, we have used 4 Å. For the calculations hereafter these structure alignment cutoff distances are used. Within this cutoff distance the residue pairs are aligned from the sequence match and are considered to be substitutions.

**Fig. 5:**
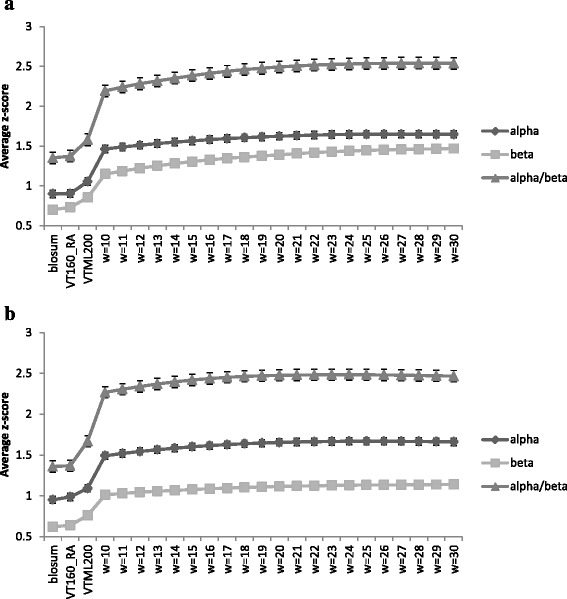
**a** The average z-scores obtained for Blosum62, VT160_RA, and VTML200 matrices, and for different weights of combined topology-based matrices obtained using Blosum62 as the basis matrix. The results are shown for helical, beta sheet, and mixed alpha and beta classes. **b** The average z-scores obtained for Blosum62, VT160_RA, and VTML200 matrices, and different weights of combined topology-based matrices obtained using VT160_RA as the basis matrix. Results are shown for helical, beta sheet, and alpha/beta classes. Z-scores are averaged over all the topologies for each weight for each class. Only improved cases are used to obtain averages. All the z-scores obtained for both improved and other cases are shown in Additional file 1: Tables S4 (a), (b) and (c). The average z-scores for all topologies and the improved topologies are compared in Additional file 1: Table S5. There is no significant difference in z-scores when the weights are changed. It can be seen that the z-scores level off with weight but when the weights are more than ~ 40, z-scores rapidly decrease (not shown)

For the selected cutoff distances, the average z-scores obtained for each class with different weight coefficients are shown in Fig. 5. The z-scores are averages over all the topologies belonging to each class. The z-scores obtained using the generic matrices are also shown on the left sides for comparison. Figure 5(a) shows the average z-scores when topology-based matrices are obtained with Blosum62 as the basis matrix and Fig. 5(b) shows the z-scores when the topology matrices are based on the VT160_RA matrix. There is no significant difference in z-scores when the weights are changed. It can be seen that z-scores level off with increasing weight but when the weights are greater than ~ 40 the z-scores rapidly decrease (not shown). All results shown are for test set A. Similar results are obtained for the other two test sets (B and C). The improvement using the topology-based matrices is significant and can be seen in comparison to the generic matrices on the left side. Z-scores obtained for helical matrices are higher with respect to beta sheet and alpha/beta classes. Beta sheet shows the lowest z-score values for all matrices.

Tables 1, 2, and 3 show the average z-scores obtained for the three classes when the three generic matrices (Blosum62, VT160_RA, and VTML200) and the topology-based matrices are used. The maximum improvement obtained for each topology when the corresponding topology matrix is used is also shown for topology matrices obtained by using Blosum62 as the basis matrix and VT160_RA as the basis matrix. Here only the topologies that show improvement in z-score for both Blosum62 combined and VT160_RA combined topology-based matrices are shown. The z-scores obtained for all the topologies for the three datasets are given in Additional file 1: Tables S4(a), (b) and (c). The average values of z-scores of all the topologies are compared to the averages obtained only using the topologies where z-score improvements were shown in Additional file 1: Table S5.

**Table 1:** Z-scores for all helical class topologies in test set A: scores obtained for each topology for Blosum62, VT160_RA, and VTML200 matrices are shown in columns 2, 3, and 4

	Z-score						
Topology	Blosum62	VT160_RA	VTML200	Blosum62 combined		VT160_RA combined	
				Max z-score	Max % improvement	Max z-score	Max % improvement
1.10.10	1.24	1.43	1.23	2.37	65.8	2.37	66.10
1.10.30	1.19	1.33	1.21	1.90	42.5	1.95	46.11
1.10.150	1.28	1.41	1.30	2.42	71.4	2.44	72.17
1.10.238	0.73	0.88	0.77	1.42	61.0	1.46	65.87
1.10.260	0.65	0.68	0.66	1.04	52.1	1.03	51.36
1.10.375	0.96	1.10	0.97	1.54	40.3	1.55	40.85
1.10.490	1.29	1.46	1.33	1.84	26.1	1.86	27.15
1.10.533	1.00	1.05	1.03	1.60	52.5	1.60	52.85
1.10.555	1.47	1.87	1.46	2.74	46.8	2.77	48.21
1.10.565	0.89	1.44	1.02	1.88	29.9	1.91	32.21
1.10.600	1.72	2.21	1.66	2.79	25.9	2.76	24.83
1.10.620	2.60	3.09	2.76	3.11	0.8	3.11	0.88
1.10.760	0.49	0.54	0.51	0.56	3.9	0.56	4.12
1.10.1170	0.37	0.41	0.38	1.82	339.8	1.75	322.87
1.10.1200	0.95	1.03	0.99	1.13	10.1	1.17	13.22
1.10.1220	0.94	1.04	0.98	1.07	3.4	1.11	6.46
1.10.1300	3.34	3.60	3.32	3.64	1.3	3.60	0.24
1.10.3210	0.75	0.80	0.72	1.35	69.4	1.37	71.22
1.20.20	1.68	1.85	1.66	2.81	51.5	2.83	52.81
1.20.920	1.68	1.88	1.69	3.28	74.5	3.29	74.93
1.20.1070	2.85	3.32	2.84	3.66	10.3	3.67	10.52
1.20.1250	0.79	0.78	0.79	1.13	43.4	1.11	40.92
1.25.40	0.76	1.11	0.61	2.54	129.3	2.50	125.62

Columns 5 and 6 show the maximum z-score obtained and the maximum percent improvement obtained when using Blosum62 combined with topology-based matrices. Columns 7 and 8 show the show the maximum z-scores obtained and the maximum percentage improvements obtained when using VT160_RA combined with topology-based matrices. Maximum percent improvement is the improvement of combined topology-based matrices over the maximum z-score giving generic matrix for each topology. Only the topologies where z-score improvements were observed for both Blosum62 combined and VT160_RA combined matrices over all three of the generic matrices are shown

**Table 2:** Z-scores for all beta sheet class topologies in test set A: scores obtained for each topology with Blosum62, VT160_RA, and VTML200 matrices are shown in columns 2, 3, and 4

	Z-score						
Topology	Blosum62	VT160_RA	VTML200	Blosum62 combined		VT160_RA combined	
				Max z-score	Max % improvement	Max z-score	Max % improvement
2.10.60	1.10	1.08	1.10	2.37	114.58	2.34	112.47
2.30.29	1.33	1.66	1.39	2.69	61.59	2.71	62.86
2.30.42	1.57	1.58	1.54	1.82	15.25	1.82	15.63
2.30.110	0.06	−0.12	−0.12	0.55	747.16	0.46	612.32
2.40.70	1.45	1.58	1.39	2.41	52.96	2.34	48.40
2.40.128	1.33	1.56	1.34	1.82	16.53	1.82	16.18
2.40.320	0.56	0.10	0.32	0.77	37.95	0.60	8.80
2.60.40	0.76	0.73	0.68	1.18	53.96	1.16	52.30
2.70.40	2.24	2.65	2.25	3.05	15.35	3.04	14.99
2.102.10	0.47	0.59	0.49	0.86	46.98	0.94	59.66
2.170.10	−0.07	−0.09	−0.20	1.19	1719.11	1.11	1613.02

Columns 5 and 6 show the maximum z-scores obtained and the maximum percentage improvements obtained when using Blosum62 combined with topology-based matrices. Columns 7 and 8 show the show the maximum z-scores obtained and the maximum percentage improvements obtained when using VT160_RA combined with topology-based matrices. Maximum percent improvement is the improvement of combined topology-based matrices over the maximum z-score giving generic matrix for each topology. Only the topologies where z-score improvements were observed for both Blosum62 combined and VT160_RA combined matrices over all three of the generic matrices are shown.

**Table 3:** Z-scores for mixed alpha and beta class topologies in test set A: scores obtained for each topology for Blosum62, VT160_RA, and VTML200 matrices are shown in columns 2, 3, and 4

	Z-score						
Topology	Blosum62	VT160_RA	VTML200	Blosum62 combined		VT160_RA combined	
				Max z-score	Max % improvement	Max z-score	Max % improvement
3.10.20	2.13	2.36	2.17	2.40	2.03	2.39	1.51
3.10.100	3.01	3.67	3.21	5.52	50.48	5.57	51.77
3.10.120	2.78	2.85	2.65	4.14	45.17	4.13	44.83
3.10.129	1.06	1.06	1.11	1.26	13.65	1.14	3.03
3.10.130	2.26	2.62	2.31	4.58	74.78	4.55	73.84
3.10.180	1.62	1.70	1.64	2.58	51.77	2.58	51.89
3.30.30	2.70	3.07	2.75	3.70	20.80	3.75	22.31
3.30.70	2.38	2.73	2.41	3.71	35.56	3.70	35.54
3.30.420	0.14	0.27	0.29	0.29	1.15	0.30	3.54
3.30.428	2.35	2.84	2.47	3.18	11.93	3.26	14.70
3.30.465	2.03	2.11	2.02	2.77	31.30	2.77	31.20
3.30.505	2.52	2.91	2.71	3.13	7.45	3.21	10.27
3.30.1050	2.87	3.16	2.74	3.47	9.85	3.34	5.55
3.30.1520	2.37	2.78	2.48	2.86	2.77	2.82	1.47
3.40.20	3.42	3.81	3.46	4.55	19.33	4.55	19.21
3.40.33	3.05	3.36	2.94	4.90	45.88	4.92	46.64
3.40.109	0.72	0.91	0.29	1.49	64.71	1.46	60.88
3.40.140	4.38	4.79	4.48	5.13	7.03	5.19	8.38
3.40.718	0.42	1.00	0.81	2.75	175.14	2.76	176.14
3.40.980	2.79	3.27	2.85	3.75	14.65	3.81	16.45
3.40.1050	0.07	0.32	0.02	2.23	595.27	2.14	567.18
3.60.21	0.92	0.84	0.79	1.69	83.39	1.67	80.72
3.70.10	3.29	3.77	3.29	4.34	15.15	4.31	14.41
3.90.79	1.86	2.05	1.84	3.01	46.42	2.89	40.90
3.90.730	5.25	5.62	5.34	5.69	1.34	5.67	1.01

Columns 5 and 6 show the maximum z-scores obtained and the maximum percentage improvements obtained when using Blosum62 combined with topology-based matrices. Columns 7 and 8 show the maximum z-scores obtained and the maximum percentage improvements obtained when using VT160_RA combined with topology-based matrices. Maximum percentage improvements are the improvements of combined topology-based matrices over the maximum z-score giving generic matrix for each topology. Only the topologies where z-score improvements were observed for both Blosum62 combined and VT160_RA combined matrices over all three of the generic matrices are shown.

The percent number of improvements obtained for the three classes are shown in Table 4. The average percentage of improvement obtained is 61 %. For the Blosum62 combined topology-based matrices, all helical class improvements are observed for 73 % of the cases, all beta sheet class 53 % and for alpha/beta 56 %. For VT160_RA combined topology-based matrices, all helical class show improvements again for 73 % of cases and for beta sheet class 49 % and for alpha/beta 56 %. Overall the number of improvements is slightly higher for the topology-based matrices obtained using Blosum62 as the basis matrix. And the best results are obtained for all helical topologies.

**Table 4:** Average percentage of the number of topologies showing improvements for the combined topology-based matrices compared with the three generic matrices (Blosum62, VT160_RA, and VTML200)

	% showing improvements	
	Blosum62 combined	VT160_RA combined
All helical	73	73
All beta sheet	53	49
Mixed alpha and beta^a^	56	56

The percentage improvements are shown for the Blosum62 combined topology-based matrices and the VT160_RA combined topology-based matrices. The results are shown for all helical, all beta sheet and mixed alpha and beta classes. Both types of combined topology-based matrices perform nearly equally well. The topology-based combined matrices show their best performances for all helical class

^a^Mixed alpha and beta corresponds to the combined group of alpha + beta structures and alpha/beta structures (consistent with CATH)

**Fig. 6:**
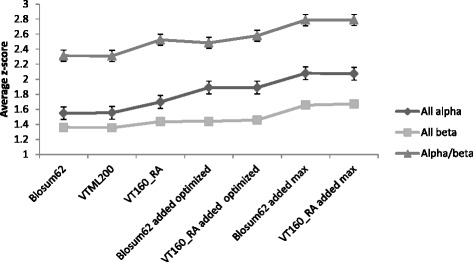
The average z-scores over all topologies in all helical, all beta sheet, and mixed alpha and beta test cases. (Blosum62 added means Blosum62 combined topology-based matrix and VT160_RA added means VT160_RA combined topology-based matrix) Optimized z-scores are the z-scores corresponding to the weight that gives the best average z-score for each class. Maximum z-score is the best (maximum) z-score obtained for each topology. In other words, optimized average z-scores have used a single weight for the whole class but the maximum average z-scores allow different weights for each topology. The z-score for the remote homology detection when using generic matrices such as Blosum62, VTML200, and VT160_RA can be further improved when structure information is incorporated into substitution matrices

Figure 6 compares the average z-scores obtained for the all helical, the all beta sheet and the alpha/beta classes for different generic matrices and topology-based matrices. The average z-scores obtained for topology-based matrices are always higher than for the standard matrices (Blosum62, VTML200 and VT160_RA). The best weight for each class is taken and is used to obtain the optimized z-score for each topology in that class. This weight is found by averaging all the z-scores obtained for each weight of each topology in the class and taking the maximum average z-score given weight for that class. The maximum z-scores are the best z-scores for each individual topology. Figure 6 shows that the maximum average z-score obtained for each class is higher than the average z-scores for the optimized weight for each class (optimized z-score). The z-score corresponding to this weight is used for all the topologies in each structural class and is termed the optimized z-score for each topology. The average z-scores for matrices obtained on the Blosum62 combined topology-based matrices and the VT160_RA combined topology-based matrices are approximately the same. This is true for both the optimized z-score and the maximum average z-score. Maximum average z-scores obtained for the topology-based matrices show a significant improvement in comparison with the standard matrices.

The newly developed structure based matrices were also compared with other structure based matrices in literature. As previously described z-scores were calculated for topology based matrices and other structure based matrices BC0030, JOHM, and PRLA1. For some cases the performance of topology based matrices clearly outperformed all three of these single matrix based matrices. For example, for all beta topologies 2.170.16 and 2.30.110 the improvements were over 20 % and for topology 2.102.10 topology the improvement was almost 50 %. Traditional Psi-blast program picks the structurally similar pairs for 23/27 of the all alpha test cases, 16/19 of the all beta test cases and 42/46 of alpha beta test cases (see Additional file 1). Matching pairs were not found for topologies 1.10.533, 1.10.1200, 1.10.1220, 1.20.1250, 2.30.30, 2.40.128, 2.60.40, 3.10.20, 3.30.420, 3.40.630 and 3.90.550. For corresponding HMM profiles generated for topologies, matching pairs were found for 23/27 all alpha test cases, 15/19 all beta test cases, and 41/46 alpha beta test cases. Matching pairs were not identified for topologies 1.10.150, 1.10.238, 1.20.120, 1.25.10, 2.30.30, 2.40.50, 2.60.40, 2.60.120, 3.10.20, 3.20.20, 3.30.70, 3.30.450, and 3.40.630. There were 2 all beta topologies (2.30.30, 2.60.40) and 2 alpha beta topologies (3.10.20, 3.40.630) for which matching pairs were not identified by either of the two conventional methods psi-blast search and HMM profile search (Table 5). Table 5 shows the e-values of hits for PSI-blast search, profile HMM search and topology based PSI-blast search for topologies that didn’t give hits for Psi-blast or profile HMMs. The topology based Psi-blast clearly outperforms these popular conventional methods for not only these cases where hits were not obtained by conventional methods but also picks the right match for all the cases we tested. All results are included in Additional file 1.

**Table 5:** The e-values of hits for PSI-blast search, profile HMM search and topology based PSI-blast search for topologies not yielding hits for PSI-blast, profile HMMs or both

	E-values		
Topology	Psi-blast	HMM	Topology psi-blast
1.10.150	1.00E-06	no hit	8.00E-18
1.10.238	3.00E-03	no hit	7.00E-38
1.10.533	no hit	3.30E-17	1.00E-30
1.10.1200	no hit	2.80E-05	5.00E-11
1.20.1250	no hit	1.10E-20	2.00E-44
2.40.128	no hit	3.00E-19	2.00E-59
2.60.40	no hit	no hit	1.00E-35
3.10.20	no hits	no hit	9.00E-25
3.30.420	no hit	5.00E-04	1.00E-48
3.30.450	3.00E-31	no hit	2.00E-52
3.40.630	no hit	no hit	1.00E-40
3.90.550	no hit	1.30E-06	1.00E-68

The topology based PSI-blast clearly outperforms these popular conventional methods for not only cases where hits were not obtained by conventional methods but for all the cases tested (for results see Additional file 1)

## Conclusions

Similarity matrices are used extensively in many different applications in computational biology. Standard matrices like Blosum62 have been generated without taking into account any topological information, although the statistics of amino acid substitutions vary with protein topology. There are some amino acid substitutions that occur more frequently in some topologies than in others and these are usually substitutions do not affect the function of these proteins. The hypothesis for this study has been that different protein topologies exhibit different amino acid substitution statistics. Here we have used structural alignment of protein structures belonging to each CATH topology and used these alignments to develop similarity matrices for each CATH topology by making amino acid substitution assignments directly from the structure alignments. We combine this structural information with general purpose matrices so that both sequence and structure components are incorporated into the newly generated topology specific matrices. We have tested our matrices using a dataset of distant homologous proteins that belong to unique CATH topologies. Our results show improved performances in sequence matching when we use our new combined topology-based matrices compared to using generic standard matrices such as Blosum62. Our combined topology-based matrices were able to distinguish structurally similar protein pairs with a better fidelity than the generic standard matrices such as Blosum62, VT160_RA and VTML200.

We have used generic matrices as anchor matrices for our topology-based matrices by adding these as perturbations to the standard matrices. This is specifically important for cases where there are too few structures in a protein family. It is evident from the scores obtained using VTML200 as the basis for generating combined topology-based matrices in the preliminary dataset that the improvements observed are approximately similar to those using VT160_RA. Therefore, only the Blosum62 and VT160_RA generic matrices were used for generating the combined topology-based matrices. We have used the same gap penalties (gap opening penalty of ten and gap extension penalty of 1). However, gap penalties might possibly be optimized to improve alignments to obtain further improvements. Z-scores are best when the Blosum62 matrix is used as the basis for the combined topology-based matrices compared to the scores obtained by the combined topology matrices generated using VTML160_RA as the basis.

The best weight for each class is taken and is used to obtain the optimized z-score for each topology for that class. Maximum z-scores are obtained for the corresponding weight that gives the best z-scores for each individual topology. Maximum z-scores that were obtained are always higher than the z-scores obtained with the optimized weights. This shows that there may still be room for improvement to sequence alignment. Although there is not a significant difference between the two types of topology-based matrices, slightly better results are seen for topology-based matrices built upon Blosum62 as the basis matrix. Improvements of z-scores obtained using topology-based matrices are significant compared to the scores obtained using any of the generic standard matrices. This demonstrates the importance of using topology-based similarity matrices when performing sequence matching. Sequence matching can be improved significantly by using the fold-specific similarity matrices, and this will aid in improving many aspects of homology modeling of proteins and gene annotation.

For the all helical cases the number of improvements observed is 73 % for both types of combined topology-based matrices. The number of improved cases for the beta sheet class is lower than for the helical class or the alpha/beta class. In almost any protein structure prediction the accuracy for alpha helical structures is nearly always higher than for beta sheet structures. This could be due to the fact that there are more long range interactions in beta sheet structures relative to short range interactions than in alpha helices. On average for the three classes, the original z-scores obtained for the generic matrices are doubled when topology-based matrices are used.

We have also repeated our calculations taking into account less detail than at the topology level. That is the second level of CATH (architecture level). However, we found that the discrimination power of the matrices decreases substantially. Hence, the best sequence matching scores are obtained when topology level matrices are used.

Results for sequence matching for Psi-blast search and topology based Psi-blast search, clearly show that replacing standard Blosum62 in Psi-blast search results in improvements in sequence matching. Topology based Psi-blast search also outperform profile based HMM for the cases tested. These results clearly show the importance of using topology specific similarity matrices instead of using standard matrices that are used in common practice today. A server for sequence matching using fold specific matrices is developed and will be made available in a subsequent publication.

## Additional file

Additional file 1: Supplementary materials. (DOCX 66 kb)

## Abbreviations

CATH: Classification of protein structures downloaded from the Protein Data Bank
HMM: Hidden Markov Models
Pdb: RCSN Protein Data Bank
