# Supplementary material for: Fold-specific sequence scoring improves protein sequence matching
Source: BMC Bioinformatics. 2016 Aug 30;17(1):328. doi: 10.1186/s12859-016-1198-z (PMC5006591; doi:10.1186/s12859-016-1198-z)
Supplement: Supplementary file 1 — Supplementary materials. (DOCX 66 kb) [file 12859_2016_1198_MOESM1_ESM.docx]

Table S1: List of the test topologies for all helical class and the sequences used: there are 3 possible sets of SSSD pairs (Sequence1-Sequence2, Sequence1-Sequence3 and Sequence2-Sequence3). Each set of sequence pairs is taken as a different set.

|  | All alpha | seq1 | seq2 | seq3 |
| --- | --- | --- | --- | --- |
| 1 | 1.10.10 | 2a61 | 1lj9 | 2vkv |
| 2 | 1.10.30 | 1ckt | 1qrv | 2lef |
| 3 | 1.10.150 | 1pk3 | 1kw4 | 1sxd |
| 4 | 1.10.238 | 1dqe | 1ow4 | 1j55 |
| 5 | 1.10.260 | 1r69 | 1lmb | 2ict |
| 6 | 1.10.375 | 1em9 | 2pxr | 1u7k |
| 7 | 1.10.490 | 1ngk | 1s69 | 1h97 |
| 8 | 1.10.533 | 3ygs | 1cy5 | 1ich |
| 9 | 1.10.555 | 1pbw | 1tx4 | 1f7c |
| 10 | 1.10.565 | 1ie9 | 1t7r | 2e2r |
| 11 | 1.10.600 | 1v4e | 1rqj | 1rtr |
| 12 | 1.10.620 | 1syy | 1mxr | 1mty |
| 13 | 1.10.630 | 2ij2 | 1jfb | 1n40 |
| 14 | 1.10.760 | 1gu2 | 1c75 | 1i8o |
| 15 | 1.10.1170 | 1jd5 | 2qfa | 1se0 |
| 16 | 1.10.1200 | 2jq4 | 1vku | 1t8k |
| 17 | 1.10.1220 | 2bj7 | 2cpg | 2hza |
| 18 | 1.10.1300 | 1taz | 1tbf | 1y2k |
| 19 | 1.10.1660 | 1r8d | 1q08 | 1q06 |
| 20 | 1.10.3210 | 2gz4 | 2pq7 | 1ynb |
| 21 | 1.20.120 | 1bbh | 2j8w | 1ls1 |
| 22 | 1.20.920 | 3d7c | 1e6i | 1eqf |
| 23 | 1.20.1070 | 1e12 | 1m0k | 1xio |
| 24 | 1.20.1250 | 1lki | 1alu | 1huw |
| 25 | 1.25.10 | 1qgr | 1wa5 | 1jdh |
| 26 | 1.25.40 | 1ot8 | 1wdy | 1hh8 |
| 27 | 1.50.10 | 2ri9 | 1x9d | 1dl2 |

Table S2: List of the test topologies for the all beta sheet class and the sequences used: there are 3 possible sets of SSSD pairs (Sequence1-Sequence2, Sequence1-Sequence3 and Sequence2-Sequence3). Each set of sequence pairs is taken as a different set.

|  | All beta | seq1 | seq2 | seq3 |
| --- | --- | --- | --- | --- |
| 1 | 2.10.60 | 1tgx | 2j8b | 1f94 |
| 2 | 2.30.29 | 2i5f | 1unq | 1eaz |
| 3 | 2.30.30 | 2iim | 1mhn | 1jo8 |
| 4 | 2.30.42 | 2fe5 | 1r6j | 1qau |
| 5 | 2.30.110 | 1flm | 2vpa | 1vl7 |
| 6 | 2.40.50 | 1hpc | 1qb5 | 1dcz |
| 7 | 2.40.70 | 4fiv | 2nmz | 2qp8 |
| 8 | 2.40.128 | 1lf7 | 1i4u | 2hnx |
| 9 | 2.40.160 | 3prn | 2fgq | 2por |
| 10 | 2.40.320 | 2jmu | 2fbl | 1yem |
| 11 | 2.60.40 | 1v05 | 2p49 | 1xl4 |
| 12 | 2.60.120 | 2nlr | 2dfb | 2bw8 |
| 13 | 2.70.40 | 1six | 1euw | 1pkh |
| 14 | 2.80.10 | 1ijt | 1pwa | 1q1u |
| 15 | 2.102.10 | 1rie | 1jm1 | 2b1x |
| 16 | 2.110.10 | 1gen | 1itv | 1hxn |
| 17 | 2.130.10 | 1sq9 | 1gxr | 1nr0 |
| 18 | 2.160.20 | 1pxz | 1bn8 | 1hg8 |
| 19 | 2.170.16 | 1mi8 | 1gpp | 1am2 |

Table S3: List of the test topologies for the alpha/beta class and the sequences used: there are 3 possible sets of SSSD pairs (Sequence1-Sequence2, Sequence1-Sequence3 and Sequence2-Sequence3). Each set of sequence pairs is taken as a different set.

|  | Alpha/beta | seq1 | seq2 | seq3 |
| --- | --- | --- | --- | --- |
| 1 | 3.10.20 | 2bwf | 1wm3 | 2c9w |
| 2 | 3.10.28 | 1b24 | 1m5x | 1t9i |
| 3 | 3.10.100 | 1xph | 1qdd | 1gz2 |
| 4 | 3.10.120 | 1cxy | 1cyo | 1mj4 |
| 5 | 3.10.129 | 1sh8 | 1wlu | 1s5u |
| 6 | 3.10.130 | 1onc | 1agi | 1gqv |
| 7 | 3.10.180 | 1xrk | 1kll | 1nki |
| 8 | 3.20.20 | 1ub3 | 1p1x | 1djq |
| 9 | 3.30.30 | 1bmr | 2sn3 | 1t0z |
| 10 | 3.30.40 | 1fbv | 1wfk | 1t1h |
| 11 | 3.30.70 | 1aps | 1ulr | 1vly |
| 12 | 3.30.420 | 2qxf | 2gui | 1w0h |
| 13 | 3.30.428 | 1y23 | 1fit | 1kpf |
| 14 | 3.30.450 | 1vhm | 1f5m | 1j3w |
| 15 | 3.30.465 | 2o1r | 2nqw | 2rk5 |
| 16 | 3.30.505 | 1d4t | 1lkk | 1jyr |
| 17 | 3.30.530 | 2bk0 | 1fm4 | 1jss |
| 18 | 3.30.1050 | 1c44 | 1pz4 | 1ikt |
| 19 | 3.30.1520 | 1ocs | 1xte | 1h6h |
| 20 | 3.40.20 | 1svy | 1kcq | 1f7s |
| 21 | 3.40.30 | 1r4w | 1bed | 1o8x |
| 22 | 3.40.33 | 1cfe | 1smb | 1qnx |
| 23 | 3.40.109 | 1bkj | 1nox | 1zch |
| 24 | 3.40.140 | 1r5t | 2fr5 | 1wkq |
| 25 | 3.40.225 | 1pvt | 1e4c | 1k0w |
| 26 | 3.40.430 | 1aoe | 1kmv | 3dfr |
| 27 | 3.40.630 | 1i12 | 2i6c | 1xmt |
| 28 | 3.40.710 | 2hds | 1n9b | 1yqs |
| 29 | 3.40.718 | 1w0d | 1lwd | 1cnz |
| 30 | 3.40.800 | 1gq6 | 2aeb | 1xfk |
| 31 | 3.40.850 | 1ry6 | 1f9v | 1x88 |
| 32 | 3.40.980 | 2g2c | 1uuy | 1di6 |
| 33 | 3.40.1050 | 1g5c | 1i6p | 1ekj |
| 34 | 3.40.1410 | 2p19 | 2fa1 | 2ikk |
| 35 | 3.60.10 | 2f1n | 1vyb | 1ako |
| 36 | 3.60.21 | 1s3l | 3ck2 | 1g5b |
| 37 | 3.70.10 | 1iz5 | 1rwz | 1u7b |
| 38 | 3.80.10 | 1p9a | 1ozn | 1w8a |
| 39 | 3.90.45 | 1y6h | 1xeo | 1v3y |
| 40 | 3.90.79 | 2b0v | 2b06 | 1sjy |
| 41 | 3.90.190 | 2f71 | 1lyv | 1jln |
| 42 | 3.90.226 | 1szo | 1sg4 | 1uyr |
| 43 | 3.90.550 | 1jyk | 1h7e | 1i52 |
| 44 | 3.90.730 | 1iyb | 1iqq | 1ioo |
| 45 | 3.90.850 | 1saw | 1gtt | 1nkq |
| 46 | 3.90.950 | 1vp2 | 1gtt | 1v7r |

Table S4 (a): z-scores for all alpha topologies, for the three test datasets used. The z-scores are shown for Blosum62, VT160_RA matrix, VTML200 matrix and Blosum62 added topology based similarity matrices (maximum z-score obtained for topology based matrices are shown).

|  | Z-scores for test set A | | | | Z-scores for test set B | | | | Z-scores for test set C | | | |
| --- | --- | --- | --- | --- | --- | --- | --- | --- | --- | --- | --- | --- |
| CATH ID | Blosum62 | VT160_RA | VTML200 | Topology | Blosum62 | VT160_RA | VTML200 | Topology | Blosum62 | VT160_RA | VTML200 | Topology |
| 1.10.10 | 1.24 | 1.43 | 1.23 | 2.37 | 0.71 | 0.69 | 0.63 | 0.93 | 0.57 | 0.55 | 0.54 | 1.09 |
| 1.10.30 | 1.19 | 1.33 | 1.21 | 1.71 | 0.79 | 0.77 | 0.77 | 1.01 | 0.93 | 0.89 | 0.88 | 1.32 |
| 1.10.150 | 1.28 | 1.41 | 1.30 | 2.16 | 0.96 | 1.03 | 1.02 | 1.26 | 0.97 | 0.99 | 0.94 | 1.48 |
| 1.10.238 | 0.73 | 0.88 | 0.77 | 1.42 | 0.66 | 0.61 | 0.64 | 0.38 | 0.68 | 0.64 | 0.67 | 0.50 |
| 1.10.260 | 0.65 | 0.68 | 0.66 | 0.92 | 0.80 | 0.80 | 0.83 | 1.08 | 0.67 | 0.65 | 0.69 | 0.86 |
| 1.10.375 | 0.96 | 1.10 | 0.97 | 1.54 | 0.60 | 0.73 | 0.61 | 0.72 | 0.73 | 0.93 | 0.80 | 1.28 |
| 1.10.490 | 1.29 | 1.46 | 1.33 | 1.84 | 0.73 | 0.75 | 0.77 | 0.93 | 0.54 | 0.43 | 0.43 | 1.07 |
| 1.10.533 | 1.00 | 1.05 | 1.03 | 1.48 | 0.92 | 0.91 | 0.88 | 1.02 | 0.67 | 0.70 | 0.69 | 0.97 |
| 1.10.555 | 1.47 | 1.87 | 1.46 | 2.74 | 1.98 | 2.37 | 1.95 | 3.37 | 2.30 | 2.78 | 2.44 | 3.31 |
| 1.10.565 | 0.89 | 1.44 | 1.02 | 1.88 | 3.44 | 3.71 | 3.44 | 3.43 | 2.61 | 3.34 | 2.68 | 3.11 |
| 1.10.600 | 1.72 | 2.21 | 1.66 | 2.79 | 2.98 | 3.01 | 2.52 | 2.93 | 4.59 | 4.74 | 4.58 | 4.17 |
| 1.10.620 | 2.60 | 3.09 | 2.76 | 3.20 | -1.77 | -1.22 | -1.82 | 1.53 | -1.53 | -1.02 | -1.31 | 1.50 |
| 1.10.630 | 2.51 | 2.70 | 2.54 | 2.80 | 2.60 | 2.62 | 2.41 | 2.35 | 4.78 | 4.68 | 4.77 | 3.69 |
| 1.10.760 | 0.49 | 0.54 | 0.51 | 0.51 | 1.06 | 1.13 | 1.03 | 1.56 | 0.72 | 0.70 | 0.71 | 0.89 |
| 1.10.1170 | 0.37 | 0.41 | 0.38 | 1.38 | 1.89 | 2.22 | 2.00 | 3.16 | 0.43 | 0.46 | 0.48 | 1.29 |
| 1.10.1200 | 0.95 | 1.03 | 0.99 | 1.13 | 1.14 | 1.27 | 1.15 | 1.65 | 1.36 | 1.46 | 1.41 | 2.19 |
| 1.10.1220 | 0.94 | 1.04 | 0.98 | 1.04 | 1.67 | 1.75 | 1.66 | 1.84 | 1.24 | 1.33 | 1.25 | 1.65 |
| 1.10.1300 | 3.34 | 3.60 | 3.32 | 3.82 | 4.72 | 4.80 | 4.73 | 4.31 | 4.86 | 4.85 | 4.86 | 4.17 |
| 1.10.1660 | 0.52 | 0.52 | 0.56 | 0.51 | 1.05 | 1.37 | 1.12 | 2.39 | 0.95 | 1.18 | 1.03 | 2.40 |
| 1.10.3210 | 0.75 | 0.80 | 0.72 | 1.31 | 0.44 | 0.45 | 0.45 | 1.18 | 1.19 | 1.33 | 1.07 | 2.48 |
| 1.20.120 | 1.68 | 1.85 | 1.66 | 2.80 | 0.76 | 0.79 | 0.75 | 0.50 | 0.65 | 0.62 | 0.69 | 0.28 |
| 1.20.920 | 1.68 | 1.88 | 1.69 | 2.99 | 1.18 | 1.45 | 1.20 | 3.73 | 1.63 | 1.88 | 1.70 | 3.59 |
| 1.20.1070 | 2.85 | 3.32 | 2.84 | 3.67 | 2.67 | 3.45 | 2.89 | 3.39 | 3.20 | 3.79 | 3.41 | 3.92 |
| 1.20.1250 | 0.79 | 0.78 | 0.79 | 1.13 | 0.91 | 1.15 | 0.94 | 1.68 | 0.59 | 0.62 | 0.61 | 1.45 |
| 1.25.10 | 4.21 | 4.17 | 4.23 | 4.20 | 2.80 | 2.73 | 2.78 | 2.71 | 2.50 | 2.51 | 2.35 | 2.45 |
| 1.25.40 | 0.76 | 1.11 | 0.61 | 2.54 | 0.82 | 1.04 | 0.81 | 1.65 | 0.81 | 0.90 | 0.70 | 0.66 |
| 1.50.10 | 4.62 | 4.69 | 4.62 | 4.66 | 4.05 | 4.13 | 4.04 | 3.25 | 4.75 | 4.68 | 4.74 | 3.95 |

Table S4 (b): All z-scores for obtained for all beta topologies, for the three test datasets used. The z-scores are shown for Blosum62, VT160_RA matrix, VTML200 matrix and Blosum62 added topology based similarity matrices (maximum z-score obtained for topology based matrices are shown).

|  | Z-scores for test set A | | | | Z-scores for test set B | | | | Z-scores for test set C | | | |
| --- | --- | --- | --- | --- | --- | --- | --- | --- | --- | --- | --- | --- |
| Topology | Blosum62 | VT160_RA | VTML200 | Topology | Blosum62 | VT160_RA | VTML200 | Topology | Blosum62 | VT160_RA | VTML200 | Topology |
| 2.10.60 | 1.10 | 1.08 | 1.10 | 2.37 | 1.80 | 1.96 | 1.87 | 3.28 | 1.39 | 1.69 | 1.46 | 3.11 |
| 2.30.29 | 1.33 | 1.66 | 1.39 | 2.69 | 2.49 | 2.77 | 2.54 | 3.35 | 1.80 | 2.07 | 1.92 | 2.20 |
| 2.30.30 | 1.10 | 0.92 | 0.98 | 0.86 | 1.88 | 1.95 | 1.90 | 2.02 | 1.43 | 1.39 | 1.45 | 1.30 |
| 2.30.42 | 1.57 | 1.58 | 1.54 | 1.82 | 0.91 | 0.86 | 0.91 | 2.19 | 0.93 | 0.97 | 0.96 | 1.28 |
| 2.30.110 | 0.06 | -0.12 | -0.12 | 0.55 | 0.87 | 0.75 | 0.91 | 0.61 | 0.21 | 0.28 | 0.24 | 0.00 |
| 2.40.50 | 0.67 | 0.57 | 0.63 | 0.00 | 0.63 | 0.49 | 0.66 | 0.00 | 0.73 | 0.68 | 0.77 | 0.16 |
| 2.40.70 | 1.45 | 1.58 | 1.39 | 2.41 | -2.40 | -2.44 | -2.41 | 0.00 | -2.44 | -2.53 | -2.51 | 0.00 |
| 2.40.128 | 1.33 | 1.56 | 1.34 | 1.82 | 0.58 | 0.56 | 0.58 | 0.32 | 0.39 | 0.34 | 0.41 | 0.20 |
| 2.40.160 | 2.99 | 3.12 | 3.00 | 2.51 | 3.35 | 3.43 | 3.40 | 2.64 | 3.29 | 3.17 | 3.21 | 2.36 |
| 2.40.320 | 0.56 | 0.10 | 0.32 | 0.77 | 0.07 | -0.07 | -0.03 | 0.14 | 1.03 | 1.26 | 1.03 | 1.51 |
| 2.60.40 | 0.76 | 0.73 | 0.68 | 1.18 | 0.48 | 0.54 | 0.53 | 0.56 | 0.61 | 0.64 | 0.65 | 0.79 |
| 2.60.120 | 1.91 | 2.31 | 2.03 | 1.41 | 4.03 | 4.07 | 4.05 | 3.49 | 0.60 | 0.73 | 0.50 | 1.56 |
| 2.70.40 | 2.24 | 2.65 | 2.25 | 3.05 | 0.60 | 1.07 | 0.68 | 1.43 | 1.05 | 1.27 | 1.11 | 1.47 |
| 2.80.10 | 2.10 | 2.35 | 2.09 | 2.26 | 2.63 | 2.94 | 2.67 | 2.89 | 1.34 | 1.77 | 1.43 | 1.61 |
| 2.102.10 | 0.47 | 0.59 | 0.49 | 0.86 | 0.46 | 0.67 | 0.47 | 2.11 | 1.02 | 0.89 | 1.03 | 0.92 |
| 2.110.10 | 3.48 | 3.70 | 3.53 | 3.54 | 2.71 | 3.22 | 2.88 | 3.05 | 2.63 | 3.22 | 2.81 | 3.16 |
| 2.130.10 | 1.78 | 2.06 | 2.09 | 1.95 | 2.29 | 2.13 | 2.01 | 1.54 | 3.40 | 3.09 | 3.12 | 2.11 |
| 2.160.20 | 2.69 | 2.54 | 2.58 | 2.39 | 2.82 | 2.43 | 2.67 | 2.22 | 2.08 | 1.78 | 1.93 | 2.01 |
| 2.170.10 | -0.07 | -0.09 | -0.20 | 1.19 | 0.72 | 1.23 | 0.84 | 2.01 | 1.56 | 1.73 | 1.44 | 1.59 |

Table S4 (c): z-scores for alpha/beta topologies, for the three test datasets used. The z-scores are shown for Blosum62, VT160_RA matrix, VTML200 matrix and Blosum62 added topology based similarity matrices (maximum z-score obtained for topology based matrices are shown).

|  | Z-scores for test set A | | | | Z-scores for test set B | | | | Z-scores for test set C | | | |
| --- | --- | --- | --- | --- | --- | --- | --- | --- | --- | --- | --- | --- |
| Topology | Blosum62 | VT160_RA | VTML200 | Topology | Blosum62 | VT160_RA | VTML200 | Topology | Blosum62 | VT160_RA | VTML200 | Topology |
| 3.10.20 | 2.13 | 2.36 | 2.17 | 2.40 | 0.98 | 0.92 | 0.91 | 1.02 | 0.95 | 0.92 | 0.87 | 1.45 |
| 3.10.28 | 0.34 | 0.46 | 0.43 | 0.25 | 0.45 | 0.52 | 0.42 | 0.95 | 4.19 | 4.66 | 4.19 | 4.62 |
| 3.10.100 | 3.01 | 3.67 | 3.21 | 5.52 | 2.07 | 2.58 | 2.09 | 4.92 | 3.63 | 4.12 | 3.70 | 5.21 |
| 3.10.120 | 2.78 | 2.85 | 2.65 | 4.14 | 2.98 | 3.12 | 2.93 | 4.31 | 2.24 | 2.39 | 2.19 | 3.78 |
| 3.10.129 | 1.06 | 1.06 | 1.11 | 1.26 | 1.19 | 1.05 | 1.06 | 0.63 | 1.17 | 1.04 | 1.09 | 1.51 |
| 3.10.130 | 2.26 | 2.62 | 2.31 | 4.58 | 0.99 | 1.17 | 0.98 | 3.75 | 1.51 | 1.86 | 1.69 | 3.43 |
| 3.10.180 | 1.62 | 1.70 | 1.64 | 2.58 | 1.11 | 0.98 | 1.03 | 1.46 | 0.61 | 0.66 | 0.57 | 0.69 |
| 3.20.20 | 3.50 | 4.05 | 3.17 | 3.59 | 0.88 | 0.66 | 0.82 | 1.00 | 1.00 | 1.07 | 1.07 | 1.27 |
| 3.30.30 | 2.70 | 3.07 | 2.75 | 3.70 | 1.94 | 2.06 | 1.84 | 2.20 | 1.60 | 1.81 | 1.60 | 2.83 |
| 3.30.40 | 1.27 | 1.30 | 1.31 | 1.09 | 1.28 | 1.12 | 1.22 | 1.21 | 1.36 | 1.53 | 1.33 | 1.61 |
| 3.30.70 | 2.38 | 2.73 | 2.41 | 3.71 | 1.17 | 1.14 | 1.14 | 0.94 | 1.07 | 1.00 | 1.03 | 0.73 |
| 3.30.420 | 0.14 | 0.27 | 0.29 | 0.29 | 0.35 | 0.13 | 0.48 | 0.18 | 1.17 | 2.02 | 1.50 | 1.93 |
| 3.30.428 | 2.35 | 2.84 | 2.47 | 3.18 | 0.56 | 0.87 | 0.51 | 2.66 | 0.87 | 1.08 | 0.92 | 1.62 |
| 3.30.450 | 4.69 | 5.18 | 4.68 | 4.87 | 0.94 | 0.74 | 0.91 | 0.58 | 0.94 | 0.64 | 0.79 | 0.45 |
| 3.30.465 | 2.03 | 2.11 | 2.02 | 2.77 | 2.01 | 2.25 | 2.07 | 3.09 | 1.78 | 1.83 | 1.77 | 2.11 |
| 3.30.505 | 2.52 | 2.91 | 2.71 | 3.13 | 2.12 | 2.54 | 2.30 | 2.80 | 2.75 | 3.12 | 2.78 | 3.91 |
| 3.30.530 | 5.22 | 5.45 | 5.16 | 4.44 | 0.59 | 0.73 | 0.62 | 1.12 | 0.35 | 0.81 | 0.47 | 1.00 |
| 3.30.1050 | 2.87 | 3.16 | 2.74 | 3.47 | 3.92 | 4.35 | 3.91 | 4.95 | 2.69 | 2.96 | 2.61 | 4.09 |
| 3.30.1520 | 2.37 | 2.78 | 2.48 | 2.86 | 1.39 | 1.76 | 1.38 | 2.77 | 1.76 | 2.20 | 1.91 | 3.86 |
| 3.40.20 | 3.42 | 3.81 | 3.46 | 4.55 | 1.19 | 1.37 | 1.30 | 1.36 | 1.05 | 1.13 | 1.00 | 1.10 |
| 3.40.30 | 1.11 | 1.50 | 1.29 | 0.94 | 0.32 | 0.22 | 0.26 | -0.18 | 0.84 | 0.77 | 0.96 | 0.24 |
| 3.40.33 | 3.05 | 3.36 | 2.94 | 4.90 | 1.72 | 2.40 | 1.83 | 4.39 | 1.37 | 2.03 | 1.38 | 4.29 |
| 3.40.109 | 0.72 | 0.91 | 0.29 | 1.49 | 5.75 | 5.73 | 5.64 | 4.63 | -0.23 | -0.10 | -0.29 | 1.86 |
| 3.40.140 | 4.38 | 4.79 | 4.48 | 5.13 | 0.52 | 0.47 | 0.65 | 1.25 | 0.51 | 0.36 | 0.26 | 1.84 |
| 3.40.225 | 1.50 | 2.14 | 1.74 | 2.02 | 2.29 | 2.01 | 2.01 | 2.01 | 4.22 | 4.37 | 3.94 | 3.91 |
| 3.40.430 | 4.77 | 5.23 | 4.82 | 4.66 | 3.01 | 3.79 | 3.09 | 3.56 | 3.02 | 3.96 | 3.35 | 3.63 |
| 3.40.630 | 0.93 | 0.79 | 0.87 | 0.69 | 0.90 | 0.81 | 0.87 | -0.63 | 0.37 | 0.28 | 0.40 | -1.03 |
| 3.40.710 | 2.05 | 1.67 | 1.63 | 1.41 | 3.48 | 3.70 | 3.47 | 2.89 | 0.18 | 0.69 | 0.39 | 1.76 |
| 3.40.718 | 0.42 | 1.00 | 0.81 | 2.75 | 6.16 | 6.08 | 6.05 | 4.91 | 2.54 | 2.59 | 2.22 | 3.14 |
| 3.40.800 | 4.78 | 4.83 | 4.42 | 3.99 | 4.03 | 3.80 | 3.68 | 2.93 | 3.42 | 3.38 | 3.19 | 2.46 |
| 3.40.850 | 5.86 | 5.69 | 5.82 | 4.81 | 5.70 | 5.58 | 5.62 | 4.53 | 5.81 | 5.59 | 5.72 | 4.62 |
| 3.40.980 | 2.79 | 3.27 | 2.85 | 3.75 | 0.40 | 0.69 | 0.46 | 2.32 | 0.84 | 1.06 | 0.65 | 2.28 |
| 3.40.1050 | 0.07 | 0.32 | 0.02 | 2.23 | -0.15 | -0.08 | -0.21 | 2.34 | 4.60 | 4.76 | 4.33 | 3.93 |
| 3.40.1410 | 3.50 | 3.95 | 3.54 | 3.89 | 2.91 | 3.16 | 2.81 | 3.12 | 2.37 | 2.87 | 2.60 | 2.58 |
| 3.60.10 | 2.07 | 1.99 | 2.32 | 1.76 | 1.61 | 2.18 | 1.75 | 1.68 | 0.69 | 2.33 | 1.24 | 2.48 |
| 3.60.21 | 0.92 | 0.84 | 0.79 | 1.69 | -0.19 | -0.06 | -0.12 | 1.43 | -0.10 | 0.00 | -0.09 | 1.35 |
| 3.70.10 | 3.29 | 3.77 | 3.29 | 4.34 | 2.84 | 3.11 | 2.89 | 3.85 | 2.68 | 2.95 | 2.75 | 3.69 |
| 3.80.10 | 4.38 | 4.65 | 4.34 | 3.77 | 4.34 | 4.03 | 4.16 | 2.49 | 5.24 | 4.60 | 5.07 | 3.02 |
| 3.90.45 | 4.90 | 5.24 | 4.88 | 5.20 | 4.51 | 4.67 | 4.36 | 5.07 | 4.57 | 4.94 | 4.50 | 5.21 |
| 3.90.79 | 1.86 | 2.05 | 1.84 | 3.01 | 2.13 | 2.17 | 2.07 | 3.12 | 1.78 | 2.00 | 1.71 | 2.75 |
| 3.90.190 | 2.24 | 2.46 | 2.49 | 2.04 | 5.88 | 5.85 | 5.88 | 3.99 | 3.53 | 3.38 | 3.46 | 2.44 |
| 3.90.226 | 4.29 | 4.08 | 4.15 | 2.57 | -2.66 | -1.54 | -2.16 | 1.23 | -2.39 | -1.14 | -2.33 | 1.20 |
| 3.90.550 | 0.24 | 0.74 | 0.29 | 1.30 | 2.94 | 2.80 | 2.66 | 1.95 | 1.23 | 1.41 | 1.07 | 1.40 |
| 3.90.730 | 5.25 | 5.62 | 5.34 | 5.69 | 5.23 | 5.69 | 5.39 | 5.48 | 4.20 | 4.81 | 4.35 | 4.79 |
| 3.90.850 | 5.88 | 6.02 | 5.92 | 5.28 | 4.77 | 5.25 | 4.82 | 4.76 | 3.58 | 4.06 | 3.47 | 3.58 |
| 3.90.950 | 4.92 | 5.25 | 4.91 | 4.76 | 5.05 | 5.25 | 5.01 | 4.87 | 5.11 | 5.30 | 5.11 | 4.11 |

Table S5: Z-scores for alpha, beta and alpha/beta topologies, averaged over the three datasets. The z-scores are shown for Blosum62, VT160_RA matrix, VTML200 matrix and Blosum62 added topology based similarity matrices (maximum z-score). The average z-scores obtained for all the topologies in each class and the average z-scores of improved topologies for each class are compared. Percent average improvement is the percent improvement of z-score over the average of Blosum61, VT160_RA and VTML200 z-scores.

| All alpha proteins | Blosum62 | VT160_RA | VTML200 | Topology | Percent average improvement |
| --- | --- | --- | --- | --- | --- |
| Average all | 1.55 | 1.70 | 1.55 | 2.08 | 23 |
| Average for improved cases | 1.05 | 1.21 | 1.06 | 1.88 | 41 |
|  |  |  |  |  |  |
| All beta proteins | Blosum62 | VT160_RA | VTML200 | Topology | Percent average improvement |
| Average all | 1.36 | 1.44 | 1.35 | 1.66 | 17 |
| Average for improved cases | 0.77 | 0.88 | 0.77 | 1.63 | 51 |
|  |  |  |  |  |  |
| Alpha/beta proteins | Blosum62 | VT160_RA | VTML200 | Topology | Percent average improvement |
| Average all | 2.31 | 2.52 | 2.31 | 2.79 | 15 |
| Average for improved cases | 1.62 | 1.91 | 1.64 | 2.83 | 39 |

| **Table S6: The e-values of structurally similar pairs obtained by conventional Psi-blast with Blosum62, Hidden Markov Models, and psi-blast using topology based similarity matrices for all alpha topologies. The cases where no hit was obtained are shown as “no hit”.** | | | |
| --- | --- | --- | --- |
|  | **psi-blast** | **HMM** | **topology psi-blast** |
| **1.10.10** | 2.0E-06 | 2.9E-07 | 8.00E-11 |
| **1.10.30** | 2.00E-18 | 3.9E-26 | 7.00E-29 |
| **1.10.150** | 1.0E-06 | **no hit** | 8.00E-18 |
| **1.10.238** | 3.0E-03 | **no hit** | 7.00E-38 |
| **1.10.260** | 1.0E-03 | 2.9E-13 | 1.00E-16 |
| **1.10.375** | 5.0E-49 | 2.5E-33 | 6.00E-60 |
| **1.10.490** | 1.0E-43 | 2.4E-20 | 3.00E-38 |
| **1.10.533** | **no hit** | 3.3E-17 | 1.00E-30 |
| **1.10.555** | 2.0E-62 | 1.7E-74 | 2.00E-85 |
| **1.10.565** | 4.0E-93 | 1.5E-73 | e-103 |
| **1.10.600** | 5.0E-12 | 3.90E-29 | 7.00E-73 |
| **1.10.620** | e-133 | 4.00E-110 | e-149 |
| **1.10.630** | e-151 | 4.60E-130 | e-170 |
| **1.10.760** | 7.0E-23 | 1.2E-07 | 3.00E-23 |
| **1.10.1170** | 4.0E-39 | 8.5E-47 | 4.00E-91 |
| **1.10.1200** | **no hit** | 2.8E-05 | 5.00E-11 |
| **1.10.1220** | **no hit** | 6.8E-15 | 2.00E-11 |
| **1.10.1300** | e-120 | 1.0E-152 | e-171 |
| **1.10.1660** | 4.0E-24 | 1.1E-29 | 3.00E-35 |
| **1.10.3210** | 2.0E-59 | 2.0E-40 | 3.00E-66 |
| **1.20.120** | 6.0E-12 | **no hit** | 1.00E-48 |
| **1.20.920** | 7.0E-45 | 2.9E-47 | 1.00E-57 |
| **1.20.1070** | 2.0E-63 | 9.7E-63 | 4.00E-91 |
| **1.20.1250** | **no hit** | 1.1E-20 | 2.00E-44 |
| **1.25.10** | 7.6E-01 | **no hit** | 0 |
| **1.25.40** | 3.0E-14 | 1.7E-39 | 6.00E-59 |
| **1.50.10** | 0.0E+00 | 1.4E-95 | 0 |

|  | **psi-blast** | **HMM** | **topology psi-blast** |
| --- | --- | --- | --- |
| **2.10.60** | 0.57 | 4.00E-17 | 2.00E-16 |
| **2.30.29** | 3.00E-37 | 2.60E-19 | 2.00E-44 |
| **2.30.30** | **no hit** | no hit | 6.3 |
| **2.30.42** | 4.00E-21 | 7.70E-17 | 1.00E-29 |
| **2.30.110** | 1.5 | 8.10E-47 | 2.4 |
| **2.40.50** | 0.25 | **no hit** | 2.00E-43 |
| **2.40.70** | 8.00E-34 | 5.90E-07 | 5.00E-41 |
| **2.40.128** | **no hit** | 3.00E-19 | 2.00E-59 |
| **2.40.160** | 0.19 | 3.00E-60 | 9.00E-80 |
| **2.40.320** | 5.00E-48 | 5.30E-46 | 3.00E-60 |
| **2.60.40** | **no hit** | **no hit** | 1.00E-35 |
| **2.60.120** | 6.3 | **no hit** | 8.00E-69 |
| **2.70.40** | 6.00E-55 | 3.40E-43 | 1.00E-57 |
| **2.80.10** | 2.00E-11 | 2.60E-15 | 4.00E-66 |
| **2.102.10** | 3.00E-46 | 3.30E-63 | 8.00E-62 |
| **2.110.10** | 2.00E-83 | 7.10E-74 | 2.00E-87 |
| **2.130.10** | 1.00E-31 | 2.50E-46 | e-124 |
| **2.160.20** | e-110 | 5.80E-76 | e-116 |
| **2.170.16** | 2.00E-39 | 4.50E-77 | 3.00E-49 |

|  | **psi-blast** | **HMM** | **topology psi-blast** |
| --- | --- | --- | --- |
| **3.10.20** | **no hits** | **no hit** | 9.00E-25 |
| **3.10.28** | 4.00E-19 | 1.70E-41 | 3.00E-33 |
| **3.10.100** | 1.00E-49 | 9.10E-38 | 2.00E-59 |
| **3.10.120** | 9.00E-35 | 3.40E-38 | 1.00E-36 |
| **3.10.129** | 6.90E-02 | 9.50E-17 | 2.00E-31 |
| **3.10.130** | 2.00E-35 | 3.50E-50 | 1.00E-47 |
| **3.10.180** | 2.00E-36 | 1.00E-13 | 4.00E-32 |
| **3.20.20** | 4.00E-16 | **no hit** | 2.00E-14 |
| **3.30.30** | 7.00E-05 | 1.90E-15 | 9.00E-16 |
| **3.30.40** | 1.50E+00 | 1.20E-03 | 6.00E-24 |
| **3.30.70** | 1.00E-07 | **no hit** | 3.00E-09 |
| **3.30.420** | **no hit** | 5.00E-04 | 1.00E-48 |
| **3.30.428** | 2.00E-46 | 5.60E-32 | 1.00E-57 |
| **3.30.450** | 3.00E-31 | **no hit** | 2.00E-52 |
| **3.30.465** | 1.20E-01 | 1.90E-14 | 2.00E-24 |
| **3.30.505** | 1.00E-40 | 2.20E-30 | 3.00E-51 |
| **3.30.530** | 4.00E-32 | 3.60E-17 | 2.00E-18 |
| **3.30.1050** | 8.00E-61 | 8.30E-39 | 2.00E-32 |
| **3.30.1520** | 2.00E-41 | 9.10E-42 | 3.00E-43 |
| **3.40.20** | 2.00E-46 | 1.60E-26 | 2.00E-61 |
| **3.40.30** | 3.00E-64 | 7.80E-17 | 3.00E-37 |
| **3.40.33** | 5.00E-55 | 2.30E-57 | 5.00E-65 |
| **3.40.109** | 1.00E-63 | 3.10E-68 | 8.00E-60 |
| **3.40.140** | 6.00E-61 | 1.10E-37 | 4.00E-53 |
| **3.40.225** | 8.00E-74 | 6.20E-79 | 5.00E-76 |
| **3.40.430** | 2.00E-75 | 1.00E-57 | 2.00E-72 |
| **3.40.630** | **no hit** | **no hit** | 1.00E-40 |
| **3.40.710** | 4.00E-90 | 2.10E-51 | e-116 |
| **3.40.718** | e-132 | 1.10E-57 | e-113 |
| **3.40.800** | e-106 | 9.80E-104 | 2.00E-91 |
| **3.40.850** | e-118 | 1.20E-122 | e-140 |
| **3.40.980** | 6.00E-53 | 5.40E-53 | 1.00E-50 |
| **3.40.1050** | 8.00E-61 | 3.10E-80 | 2.00E-68 |
| **3.40.1410** | 1.00E-57 | 5.80E-52 | 3.00E-74 |
| **3.60.10** | 1.00E-70 | 2.40E-67 | 7.00E-58 |
| **3.60.21** | 8.00E-57 | 7.10E-19 | 2.00E-44 |
| **3.80.10** | e-109 | 7.70E-64 | e-159 |
| **3.90.45** | 1.00E-63 | 2.00E-67 | 1.00E-81 |
| **3.90.79** | 5.00E-57 | 4.80E-34 | 1.00E-42 |
| **3.90.190** | e-104 | 1.10E-52 | 2.00E-87 |
| **3.90.226** | 7.00E-85 | 5.70E-34 | 2.00E-55 |
| **3.90.550** | **no hit** | 1.30E-06 | 1.00E-68 |
| **3.90.730** | 5.00E-82 | 3.80E-79 | 3.00E-97 |
| **3.90.850** | 8.00E-80 | 5.70E-77 | 2.00E-79 |
| **3.90.950** | 2.00E-80 | 4.50E-84 | 1.00E-81 |

**Table S7: Number of structures in each CATH topology used in the building of topology based matrices for all alpha, all beta and alpha beta classes.**

| All alpha topology | Number of structures |
| --- | --- |
| 1_10_10 | 344 |
| 1_10_1170 | 7 |
| 1_10_1200 | 20 |
| 1_10_1220 | 8 |
| 1_10_1300 | 4 |
| 1_10_150 | 103 |
| 1_10_1660 | 6 |
| 1_10_238 | 83 |
| 1_10_260 | 29 |
| 1_10_30 | 9 |
| 1_10_3210 | 9 |
| 1_10_375 | 6 |
| 1_10_490 | 34 |
| 1_10_533 | 15 |
| 1_10_555 | 5 |
| 1_10_565 | 18 |
| 1_10_600 | 10 |
| 1_10_620 | 9 |
| 1_10_630 | 19 |
| 1_10_760 | 35 |
| 1_20_1070 | 11 |
| 1_20_120 | 108 |
| 1_20_1250 | 31 |
| 1_20_920 | 6 |
| 1_25_10 | 16 |
| 1_25_40 | 86 |
| 1_50_10 | 33 |

| All beta topology | Number of structures |
| --- | --- |
| 2_102_10 | 11 |
| 2_10_60 | 14 |
| 2_110_10 | 5 |
| 2_130_10 | 36 |
| 2_160_20 | 25 |
| 2_170_16 | 5 |
| 2_30_110 | 41 |
| 2_30_29 | 51 |
| 2_30_30 | 151 |
| 2_30_42 | 55 |
| 2_40_128 | 53 |
| 2_40_160 | 21 |
| 2_40_320 | 3 |
| 2_40_50 | 177 |
| 2_40_70 | 30 |
| 2_60_120 | 282 |
| 2_60_40 | 549 |
| 2_70_40 | 11 |
| 2_80_10 | 40 |

| Alpha beta  topology | Number of structures |
| --- | --- |
| 3_10_100 | 32 |
| 3_10_120 | 7 |
| 3_10_129 | 45 |
| 3_10_130 | 8 |
| 3_10_180 | 45 |
| 3_10_20 | 117 |
| 3_10_28 | 14 |
| 3_20_20 | 384 |
| 3_30_1050 | 6 |
| 3_30_1520 | 5 |
| 3_30_30 | 26 |
| 3_30_40 | 31 |
| 3_30_420 | 91 |
| 3_30_428 | 8 |
| 3_30_450 | 64 |
| 3_30_465 | 17 |
| 3_30_505 | 29 |
| 3_30_530 | 19 |
| 3_30_70 | 328 |
| 3_40_1050 | 4 |
| 3_40_109 | 14 |
| 3_40_140 | 16 |
| 3_40_1410 | 12 |
| 3_40_20 | 15 |
| 3_40_225 | 6 |
| 3_40_30 | 118 |
| 3_40_33 | 5 |
| 3_40_430 | 15 |
| 3_40_630 | 126 |
| 3_40_710 | 26 |
| 3_40_718 | 7 |
| 3_40_800 | 7 |
| 3_40_850 | 7 |
| 3_40_980 | 8 |
| 3_60_10 | 9 |
| 3_60_21 | 21 |
| 3_70_10 | 8 |
| 3_80_10 | 21 |
| 3_90_190 | 33 |
| 3_90_226 | 24 |
| 3_90_45 | 6 |
| 3_90_550 | 24 |
| 3_90_730 | 6 |
| 3_90_79 | 24 |
| 3_90_850 | 5 |
| 3_90_950 | 6 |

**Table S8: The number of topology matrices developed and the number of structures used in the structure alignments to generate matrices.**

| Secondary structure class | No of topologies | No of structures used |
| --- | --- | --- |
| All alpha | 198 | 1064 |
| All beta | 137 | 1560 |
| Alpha beta | 330 | 1849 |
